# Pyromeconic acid-enriched *Erigeron annuus* water extract as a cosmetic ingredient for itch relief and anti-inflammatory activity

**DOI:** 10.1038/s41598-024-55365-2

**Published:** 2024-02-26

**Authors:** Minkyoung Kang, Minji Kang, Tae Hee Kim, Seong Un Jeong, Sangnam Oh

**Affiliations:** 1 Department of Food and Nutrition, Jeonju University, Jeonju, 55069 Korea; 2 Hamsoapharm R&D Center, Iksan, 54524 Korea

**Keywords:** Cell biology, Health care

## Abstract

*Erigeron annuus* (EA), traditionally used to treat disorders such as diabetes and enteritis, contains a variety of chemicals, including caffeic acid, flavonoids, and coumarins, providing antifungal and antioxidative benefits. However, the ingredients of each part of the EA vary widely, and there are few reports on the functionality of water extracts in skin inflammation and barrier protection. We assessed the therapeutic properties of the extract of EA without roots (EEA) and its primary ingredient, pyromeconic acid (PA), focusing on their antihistamine, anti-inflammatory, and antioxidative capabilities using HMC-1(human mast cells) and human keratinocytes (HaCaT cells). Our findings revealed that histamine secretion, which is closely related to itching, was notably reduced in HMC-1 cells following pretreatment with EEA (0.1% and 0.2%) and PA (corresponding concentration, 4.7 of 9.4 µg/mL). Similarly, they led to a marked decrease in the levels of pro-inflammatory cytokines, including IL-1β, IL-8, IL-6, and IFN-γ. Furthermore, EA and PA enhanced antioxidant enzymes, such as superoxide dismutase (SOD) and catalase (CAT), reduced malondialdehyde (MDA) production, and showed reactive oxygen species (ROS) scavenging activity in HaCaT cells. Moreover, at the molecular level, elevated levels of the pro-inflammatory cytokines IL-1β, IL-6, TARC, and MDC induced by TNF-α/IFN-γ in HaCaT cells were mitigated by treatment with EEA and PA. We also revealed the protective effects of EEA and PA against SDS-induced skin barrier dysfunction in HaCaT cells by enhancing the expression of barrier-related proteins. Using NanoString technology, a comprehensive analysis of gene expression changes indicated significant modulation of autoimmune and inflammatory genes by EEA and PA. In summary, this study suggests that EEA and the corresponding concentration of PA as an active ingredient have functional cosmetic applications to alleviate itching and improve skin health.

## Introduction

Skin inflammation and itchiness, a persistent and distressing condition, are characterized by redness and swelling and are associated with skin diseases, such as psoriasis, eczema, and atopic dermatitis^1,2^. Scratching, in response to itching, disrupts the skin barrier, promotes inflammation, and can enter pathogens, leading to secondary bacterial infections^3^. This leads to the 'itch-scratch cycle,’ where continuous skin barrier dysfunction and weakened immune function create a cycle that leads to a decline in skin health^3–5^. Topical corticosteroids and antihistamines are commonly used to treat inflammation and itching. However, their efficacy in controlling non-histaminergic itching is limited, and prolonged use can lead to side effects, such as skin atrophy, hyperpigmentation, and telangiectasias. Therefore, it is crucial to develop plant-based medicinal treatments that can effectively manage skin health and offer safe and consistent relief from skin itching and inflammation^5–7^.

Mast cells play a crucial role in allergic reactions and inflammation by storing and releasing various mediators, including cytokines such as histamine and interferon-gamma (IFN-γ). IFN-γ is a pleiotropic cytokine that regulates multiple cellular activities, including chemokine expression and release in mast cells^8^. Histamine also exerts various immune regulatory functions by modulating the functions of monocytes, T cells, macrophages, neutrophils, eosinophils, B cells, and dendritic cells^9^. Keratinocytes, the predominant cells in the epidermis, contribute to skin hydration and regulate immune responses by producing diverse chemokines and cytokines. Skin disorders such as atopic dermatitis (AD), psoriasis, and ichthyosis, characterized by inflammation and itching, often involve a Th1/Th2 immune imbalance driven by substances secreted by mast cells and keratinocytes during the inflammatory response^10–12^. Thus, maintaining immune homeostasis and protecting the skin barrier are fundamental for preserving skin health.

*Erigeron annuus* (EA), also known as annual fleabane, has been traditionally used in the treatment of various disorders such as enteritis because of its rich chemical composition, including flavonoids and coumarins^13–16^. Recent studies have demonstrated the antioxidant and enzyme-inhibitory activities of caffeic acid and pyromeconic acid, the active compounds in EA^17^, and have identified several phenolic acids that activate the AMPK pathway and have anti-obesity properties in HFD-induced obesity mice^14,18,19^. In addition, Joo et al. suggested that a 70% ethanol extract of EA significantly inhibits tyrosinase and elastase, contributing to anti-aging properties by whitening and preventing skin wrinkling^20^. Despite these benefits, there is a lack of research on the skin health benefits of this herb in reducing inflammation. However, there are few reports on the anti-inflammatory and skin barrier-protective effects of EA water extract and pyromeconic acid (PA). Therefore, we evaluated the therapeutic properties of the extract of EA without roots (EEA) and its primary active compound, pyromeconic acid (PA), focusing on their antihistamine, anti-inflammatory, and antioxidative capabilities using HMC-1 (human mast cells) and human keratinocytes (HaCaT cells).

## Results

### Quantitative analysis of pyromeconic acid (PA) distribution in *Erigeron annuus*

**Figure 1:**
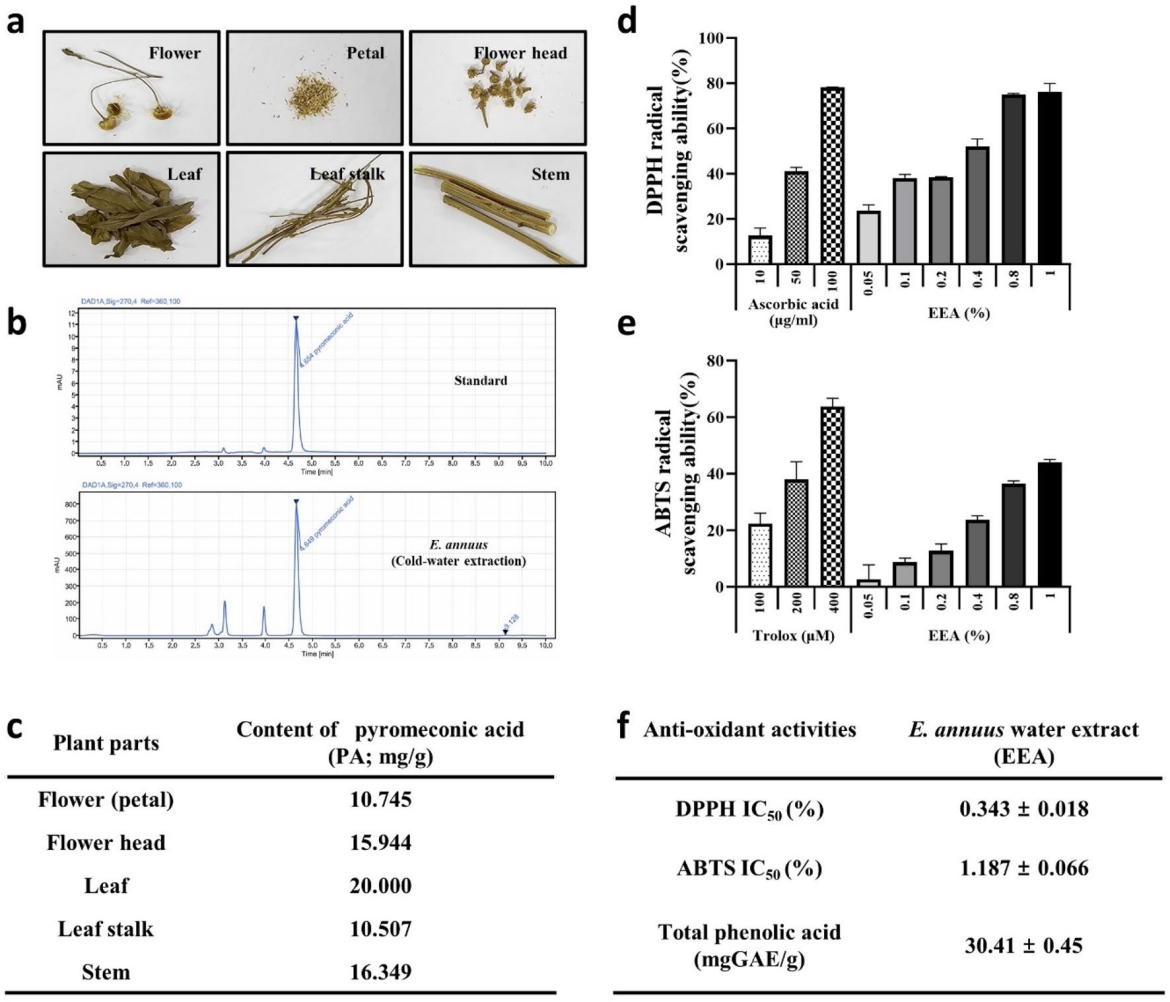
Identification of *E. annuus* water extract (EEA), DPPH free radical scavenging activity and ABTS scavenging activity. (**a**) Visual representation of distinct *Erigeron annuus* (EA) segments. (**b**) High-performance liquid chromatography (HPLC) measurement of pyromeconic acid (PA) content. (**c**) Extraction yield (%) of pyromeconic acid (PA) in different plant parts. (**d**) DPPH free radical scavenging analysis. (**e**) ABTS radical scavenging performance of the EA-water extract (EEA) and pyromeconic acid (PA). (**f**) Total phenolic and IC_50_ values for the DPPH and ABTS.

To optimize the yield of PA, we compared the PA content in different parts of *E. annuus* and evaluated various extraction methods. The levels of PA in various aboveground parts of *E. annuus* were quantitatively analyzed. These parts include the stem, leaf stalk, leaf, flower head, and flower (petals). The concentrations of PA in each component were determined as follows: flower (10.745 mg/g EA), flower head (15.944 mg/g), leaves (20.000 mg/g), leaf stalks (10.507 mg/g), and stems (16.349 mg/g) (Fig. 1a–c). These results indicate a homogenous distribution of PA in all the aboveground parts of the plant. We investigated the PA content of EEA under three different extraction conditions (Table 1). The results showed that the highest PA yield was obtained when extraction was performed using cold-water extraction at 30 °C. Therefore, to obtain the highest concentration of active compounds, EEA was obtained by water extraction at 30 °C, utilizing the entire aboveground portion of the plant, excluding the roots. These extracts were used in subsequent experiments.

**Table 1:** The pyromeconic acid content of EEA under three different extraction conditions.

Method	Solvent	Temperature (℃)	Content of pyromeconic acid (PA; mg/g)
Hot-water extraction	Distilled water	90	11.64
Cold-water extraction	Distilled water	30	41.53
Ethanol extraction	70% ethanol	30	17.77

DPPH and ABTS radical scavenging assays were performed to assess the antioxidant capacity of EEA extract. EEA and PA demonstrated substantial antioxidant activity compared with ascorbic acid. EEA was characterized by an IC_50_ value of 0.343 ± 0.018% and PA showed an IC_50_ of 15.70 ± 1.186 µg/mL (corresponding concentration, 0.33% EEA). In the ABTS assay, EEA demonstrated moderate antioxidant activity compared with Trolox, with an IC_50_ value of 1.187 ± 0.066%, and the total phenolic content in the EEA was determined to 30.41 ± 0.45 mg GAE/g, when compared to gallic acid. In addition, PA showed an IC_50_ value of 17.96 ± 0.066 µg/mL (corresponding concentration, 0.38% EEA) in the ABTS assay and was measured as 0.486 ± 0.01 mg GAE/g with compared to gallic acid (Fig. 1f). Notably, EEA exhibited an increase in DPPH and ABTS radical scavenging capacity in a dose-dependent manner, indicating its potent and progressive antioxidant efficacy (Fig. 1d,e).

### Enhanced EEA and PA antioxidant effects in H_2_O_2_-induced oxidative stress HaCaT cells

**Figure 2:**
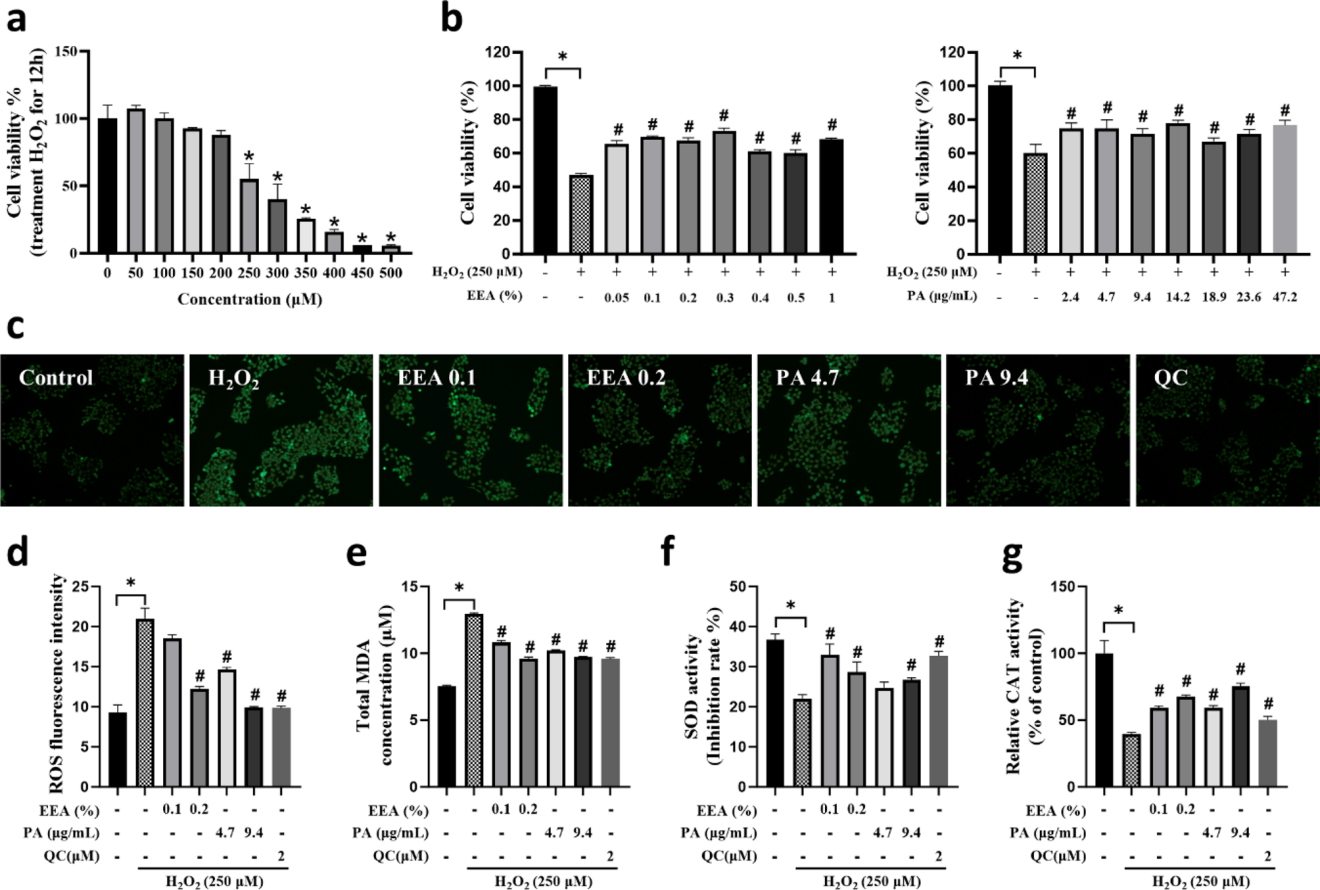
Antioxidant efficacy of *Erigeron* *annuus* water extract (EEA) on H_2_O_2_-induced oxidative stress HaCaT cells. (**a**) H_2_O_2_ induced cytotoxicity of HaCaT cells. HaCaT cells were exposed to 50–500 µM H_2_O_2_ for 12 h by MTS assay. (**b**) HaCaT cells were pretreated with EEA at different concentrations (0.05–1%) and PA at corresponding concentration (2.4–47.4 μg/mL) for 6 h prior to exposure to 250 µM H_2_O_2_ for 12 h. (**c**) HaCaT cells were pretreatment with 0.1% and 0.2% EEA, corresponding concentration 4.7 and 9.4 µg/mL PA, and positive control QC for 6 h prior to exposure to H_2_O_2_ for 12 h. ROS content was detected by DCFH-DA and examined with 20× fluorescence microscope. (**d**) The fluorescence quantitative analysis by Image J. (**e**) HaCaT cells were pretreatment with EEA and PA for 6 h prior to exposure to 250 µM H_2_O_2_. Significance: *P < 0.05 vs Control group; ^#^P < 0.05 vs H_2_O_2_-treated group.

**Figure 3:**
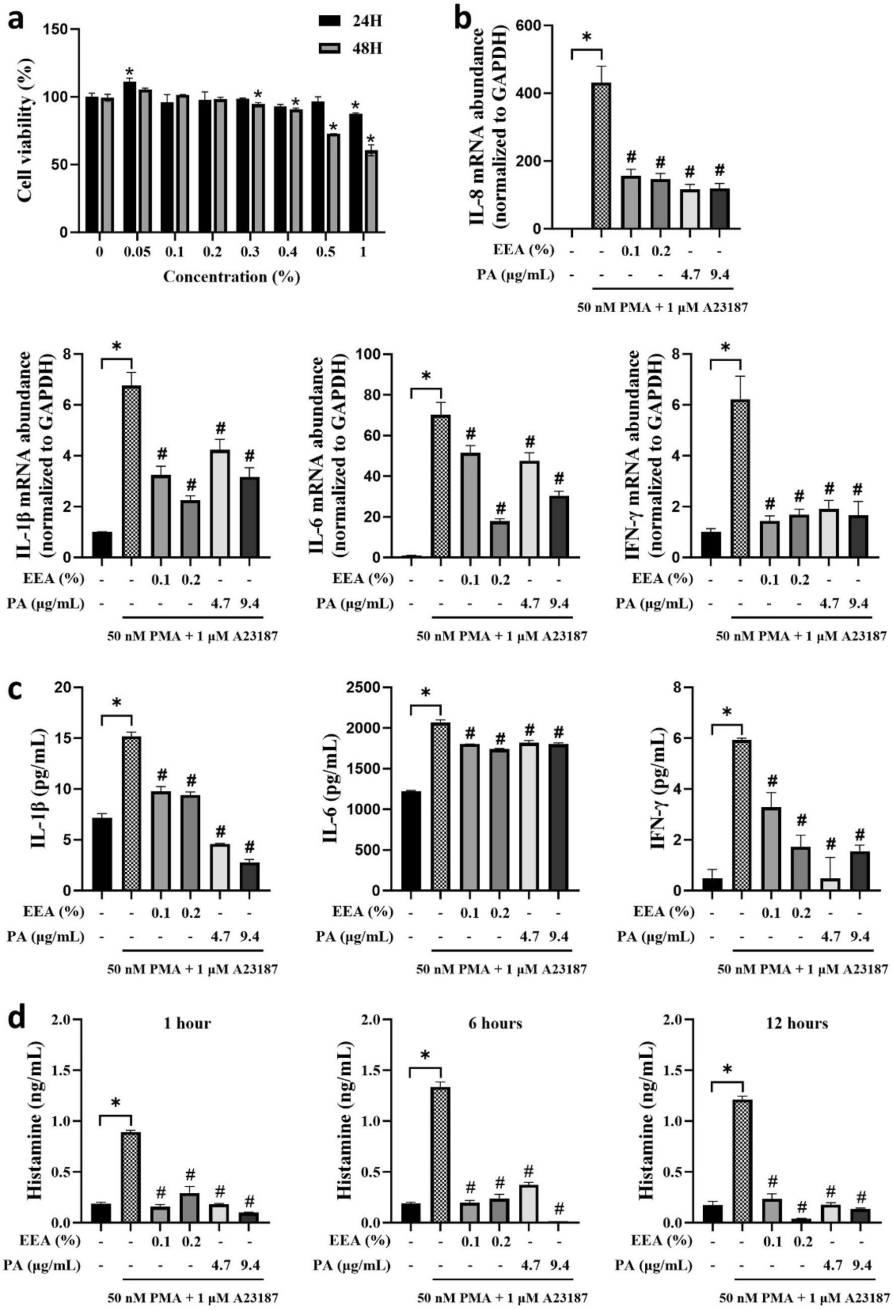
Anti-inflammatory and antihistamines efficacy of *Erigeron* *annuus* water extract (EEA) on PMACI-induced HMC-1 cells. (**a**) The MTS assay was measured. (**b**) The HMC-1 cells were pretreated with EEA (0.1% or 0.2%) and PA (respective concentration, 4.7 and 9.4 µg/mL) for 1 h and stimulated with 50 nM PMA + 1 µM A23187 for 24 h. Total RNA was extracted, and the mRNA expression of IL-1B, IL-6, IL-8, and IFN-r was measured by real-time qPCR. The protein expression (**c**) were measured using ELISA kit and (**d**) histamine level. Statistical comparisons were performed using a *t*-test (*P < 0.05. vs normal control, ^#^P < 0.05. vs PMACI group).

Antioxidants play a crucial role in managing skin diseases by neutralizing oxidative stress, which can lead to skin cell damage and inhibit mast cell degranulation, triggering histamine release and allergic reaction^21,22^. To establish an in vitro model of H_2_O_2_-induced oxidative stress, HaCaT cells were exposed to H_2_O_2_ at concentrations ranging from 50 to 500 µM. Cell viability was assessed using the MTS assay, which showed a dose-dependent decrease in viability, with 47% cell viability at 250 µM H_2_O_2_. Consequently, this concentration was used in subsequent experiments to examine the inhibitory effects of EEA and PA on oxidative stress. The EEA treatment showed that 0.05–1% concentrations significantly increased cell viability, which was dose-independent. Similarly, PA also showed a significant increase in cell viability at all concentrations corresponding to the EEA (2.4–47.2 µg/mL) (Fig. 2a,b). Cell cytotoxicity assessments of EEA in HaCaT cells (Fig. 3a) showed significant cytotoxicity at 0.4% EEA for 36 h. Thus, 0.1% and 0.2% EEA were used for further evaluation of the efficacy. The PA content at these concentrations was 4.7 µg/mL and 9.4 µg/mL, respectively. The antioxidant efficacy of EEA and PA against H_2_O_2_-induced oxidative stress in HaCaT cells was evaluated by measuring the ROS levels. Compared to the control group, the increased fluorescence signal in the H_2_O_2_-induced group confirmed the induction of oxidative stress. Pretreatment with EEA and PA significantly reduced the fluorescence signal, indicating their efficacy in reducing intracellular ROS production. In addition, 0.2% EEA and the corresponding concentration of PA showed effects similar to those of quercetin (QC), a well-known antioxidant (Fig. 2c,d).

The antioxidant enzymes, superoxide dismutase (SOD) and catalase (CAT), along with total malondialdehyde (MDA) as an indicator of oxidative damage, are established markers for assessing antioxidant efficacy^23^. Their levels were significantly altered upon induction of oxidative stress by H_2_O_2_^24^. To determine the potential of EEA and PA in alleviating H_2_O_2_-induced cellular oxidative stress, we measured total MDA and SOD and CAT activities using ELISA kits. In the H_2_O_2_-induced oxidative stress group, total MDA levels increased significantly from 0.736 ± 0.14 µM to 1.04 ± 0.52 µM compared with the control group. However, pretreatment with EEA and PA significantly reduced the total MDA levels. Simultaneously, the activities of antioxidant enzymes SOD and CAT, which were significantly decreased by H_2_O_2_ exposure, were significantly increased after pretreatment with EEA and PA (Fig. 2e–g). These results suggest the potent antioxidant properties of EEA and its active component PA.

### Inhibition of PMACI-induced cytokines and histamine responses by EEA and PA in HMC-1 cells

Histamine is central player in stimulating the development of allergic-related skin inflammatory diseases, and pro-inflammatory cytokines secreted by mast cells activate immune cells to promote an inflammatory response^9,25,26^. To investigate the antihistamine and anti-inflammatory properties of EEA and PA in HMC-1 cells, we evaluated the levels of cytokines and histamine in PMACI-stimulated HMC-1 cells. The mRNA and protein expression of the pro-inflammatory cytokines IL-8, IL-6, IL-1, and IFN-r were significantly increased by PMACI stimulation compared to the control group. However, pretreatment with EEA and PA significantly suppressed the PMACI-induced cytokine production (Fig. 3b,c). For histamine levels, we measured the effects of EEA and PA at different times of 1, 6, and 12 h of pretreatment. Notably, EEA and PA caused similar inhibition of PMACI-induced histamine production at all the evaluated pretreatment times (Fig. 3d).

### Anti-inflammatory effects of EEA and PA by inhibiting TNF-α/IFN-γ-induced proinflammatory cytokines in HaCaT cells

**Figure 4:**
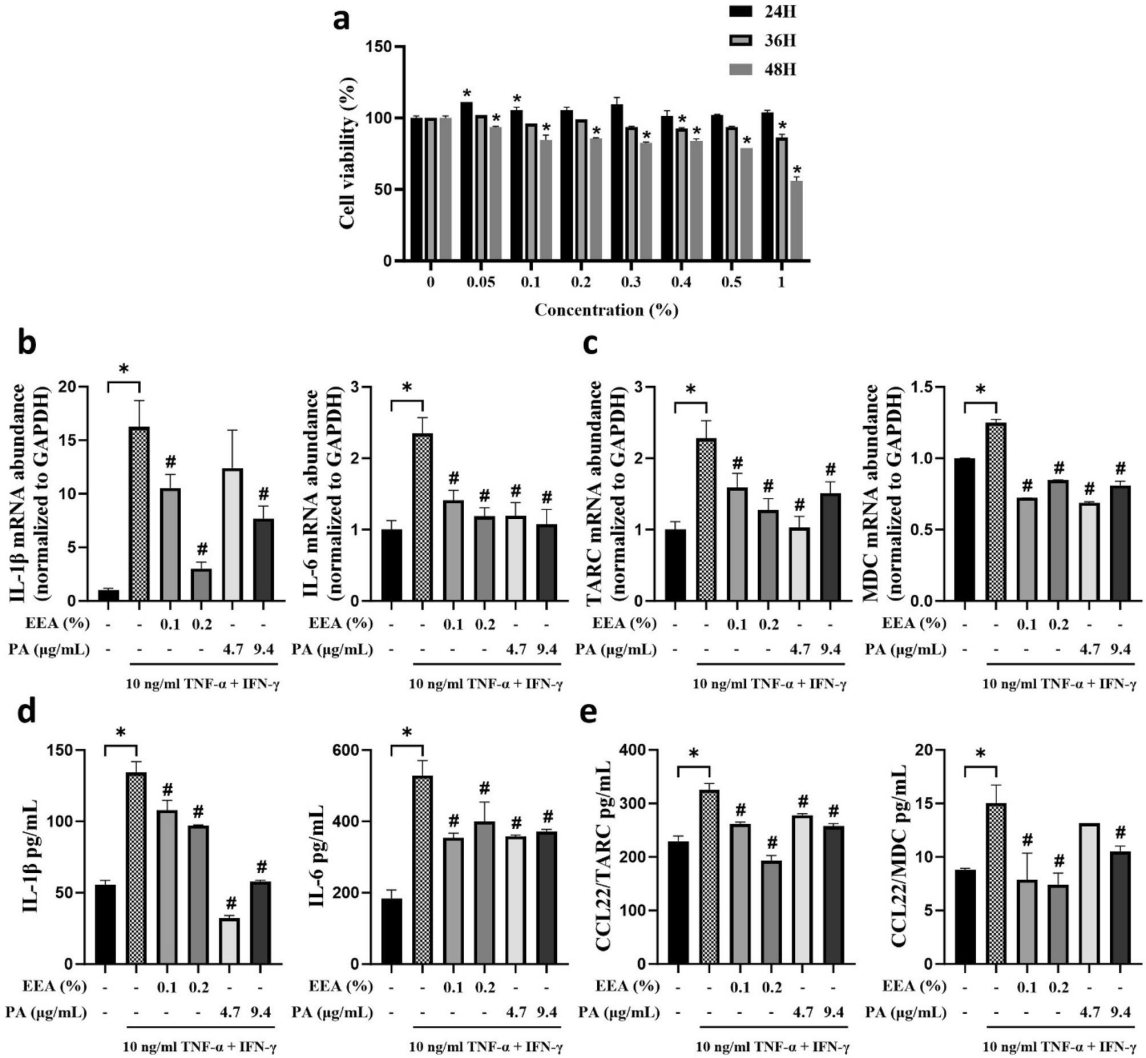
Anti-inflammatory efficacy of *Erigeron annuus* water extract (EEA) on TNF-α/IFN-γ induced HaCaT cells. (**a**) HaCaT cells were treated with EEA for 24, 36 or 48 h, and the MTS assay was performed. (**b**) The HaCaT cells were pretreated with EEA (0.1% or 0.2%) and PA (respective concentration, 4.7 and 9.4 µg/mL) for 6 h and stimulated with 10 ng/mL of T/I for 24 h. The mRNA expression of IL-1B, IL-6, TARC, and MDC was measured by RT-qPCR. (**c**) Cell supernatant was harvested 24 h after 10 ng/mL T/I stimulation, and protein expression was measured using ELISA kit.

HaCaT cells, a type of human keratinocyte, have been found to spontaneously secrete thymus and activation-regulated chemokines (TRAC) and macrophage-derived chemokines (MDC)^27,28^. These chemokines play a role in immune response and inflammation. Initially, we assessed the cytotoxicity of various concentrations of EEA in HaCaT cells. Treatment with EEA for up to 48 h significantly reduced cell viability at all the concentrations analyzed. Therefore, we limited the treatment duration to less than 48 h for efficacy testing (Fig. 4a) When HaCaT cells were stimulated with a TNF-α/IFN-γ cocktail to induce an inflammatory response, there was a notable increase in the mRNA and protein levels of inflammatory cytokines (IL-1β and IL-6) and chemokines (TARC and MDC), confirming the induction of inflammation compared with the control group. Pre-treatment with EEA and PA for 6 h substantially decreased the levels of IL1β, IL-6, TARC, and MDC (Fig. 4b–e). These findings indicate that both EEA and PA possess anti-inflammatory properties, effectively modulating inflammatory cytokine and chemokine levels in in vitro inflammation-induced HaCaT cells.

### Protective effects of EEA and PA by inhibiting SDS-induced skin barrier dysfunction in HaCaT cells

**Figure 5:**
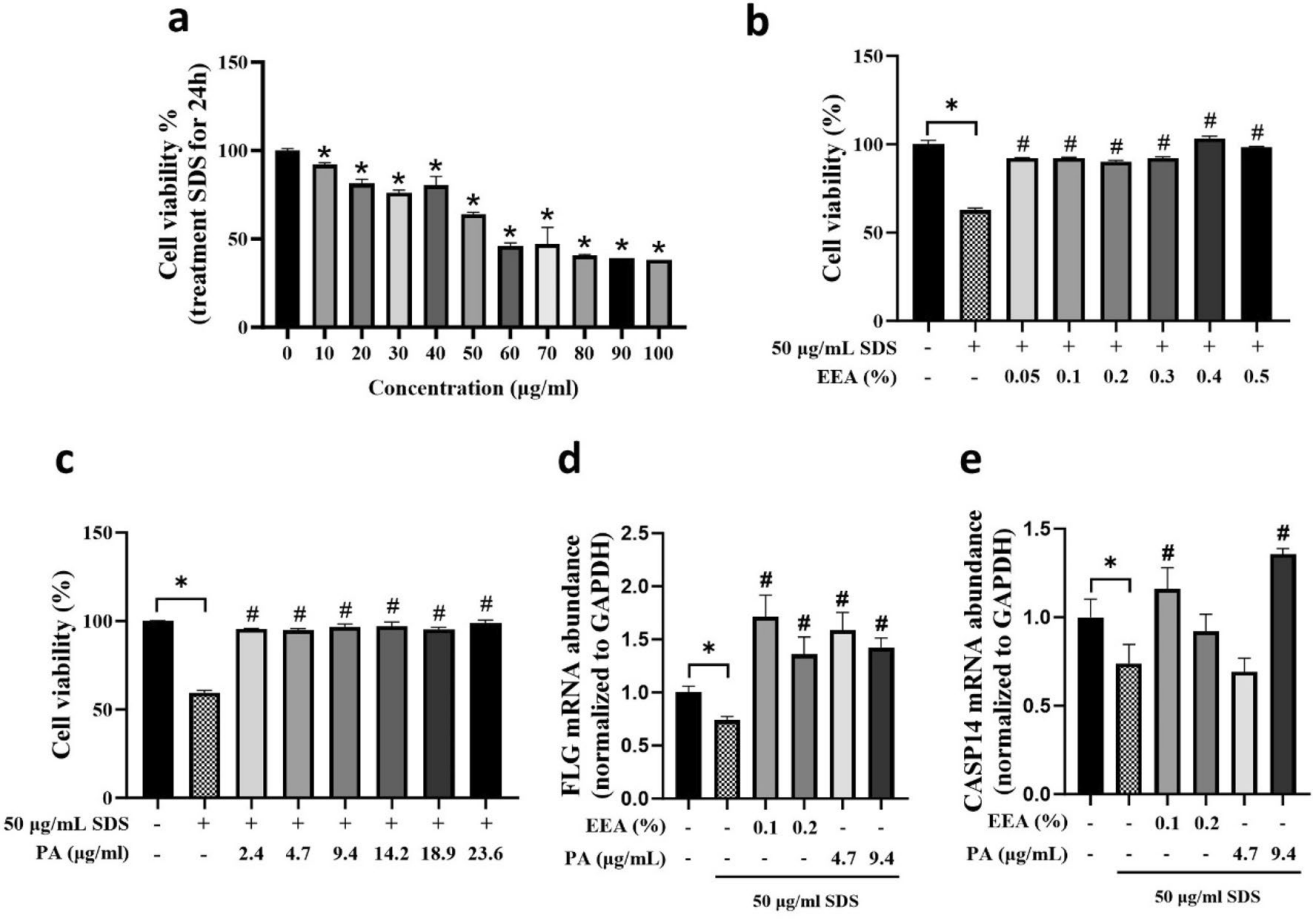
Protective effects of EEA and PA on sodium dodecyl sulfate (SDS)-induced skin barrier dysfunction in HaCaT. HaCaT cells were pre-treated with EEA or PA for 6 h and stimulated 50 µg/mL SDS for 24 after pre-treatment. (**a**) SDS induced cytotoxicity of HaCaT cells (**b**) The MTT assay was investigated of EEA. (**c**) The MTT assay was investigated of PA (**d**). The mRNA level of filaggrin (FLG) and (**e**) caspase-14 (CASP 14) were measured by real-time qPCR (**e**). *P < 0.05. vs normal control, ^#^P < 0.05. vs stimulation group.

Sodium dodecyl sulfate (SDS) is used to induce skin barrier dysfunction in HaCaT cells, a human keratinocyte line. Wenyu Ding et al. found that SDS treatment led to changes in the expression of several genes related to the skin barrier, including aquaporin3 (AQP3), filaggrin (FLG), and caspase-14 (CAP14)^29^. To determine the protective effects of EEA and PA on sodium dodecyl sulfate (SDS)-induced skin barrier dysfunction in HaCaT cells, a cell viability assay was performed. Various concentrations of SDS were used to determine the optimal concentration of stimulant. Cytotoxicity was observed at 10 μg/mL, with cell viability decreasing to 64% at 50 μg/mL and falling below 50% at 60 μg/mL SDS (Fig. 5a). We also investigated the protective effects of EEA and PA in SDS-damaged HaCaT cells. Cells were pretreated with EEA and PA and then exposed to 50 μg/mL SDS. Both EEA and PA showed significant protective effects against SDS-induced cytotoxicity at all concentrations examined. The treatment groups exposed to EEA and PA showed cell proliferation rates similar to those of the control group (Fig. 5b,c). Regarding the gene expression associated with skin barrier protection, a significant increase in caspase 14 (CASP 14) levels was observed only in the group treated with 0.1% EEA and 9.4 μg/mL PA. Conversely, filaggrin (FLG) expression was upregulated in response to both EEA and PA treatments (Fig. 5d,e). In conclusion, EEA and PA showed cytoprotective effects by increasing the expression of skin barrier-related proteins (FLG and CASP14) in HaCaT cells, which attenuated SDS-induced skin barrier damage.

### Modulation of autoimmune and inflammatory gene expression by EEA and PA in HMC-1 and HaCaT cells

NanoString technology was employed to analyze the auto-immune profiling panel and host response panel, providing a comprehensive gene expression profile. In our study, we closely monitored the expression of 786 genes in human mast cells (HMC-1) to elucidate the effects of pretreatment with EEA and PA, and PMACI induction of inflammatory conditions on gene expression. Among these genes, 639 showed statistically significant changes in expression in PMACI-positive controls compared with normal controls. We observed that 411, 414, 362, and 350 genes showed statistically significant expression changes compared to PMACI-positive controls in the EEA-and PA-treated groups, respectively.

Notably, the percentage of genes upregulated by over 1.2 fold change in the PMACI-positive control accounted for 55% of the total significant changes, whereas downregulated genes accounted for only 10%. In contrast, the EEA- and PA-treated groups showed a different pattern compared to the PMACI group: 0.1% EEA; 40%, 0.2% EEA; 23%, 4.7 μg/mL PA (PA(L)); 27% and 9.4 μg/mL PA (PA(H)); 21% of genes were upregulated and 33%, 30%, 31%, and 29% were downregulated, respectively.

**Figure 6:**
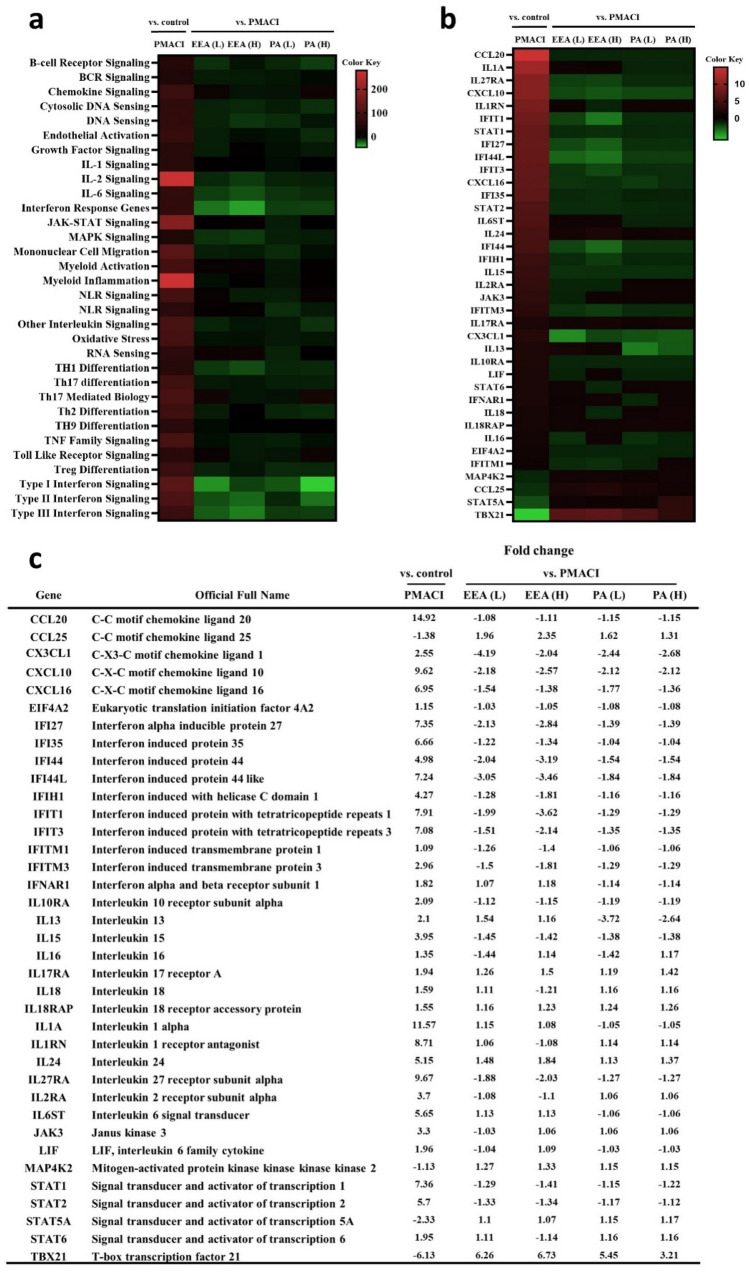
Pathway scoring and gene-set analyses of HMC-1 cells. (**a**) 38 genes identically expression in each sample. (**b**) Important pathways between the EEA and PA compared with PMACI groups. PMACI group vs baseline control group. (**c**) Organized into gene expression table.

We screened for genes with statistically significant changes and focused on those with common trends between EEA and PA to investigate how EEA and PA regulate autoimmune and host response-related genes. The heatmap clearly shows that PMACI induction primarily upregulated networks and genes associated with general inflammation (Fig. 6a,b). However, these levels were significantly attenuated by treatment with EEA and PA. Notably, the PA(H) treatment resulted in the highest proportion of downregulated genes. Both EEA and PA treatment effectively reduced the overall chemokine and cytokine levels and significantly reduced interferon-related genes. TBX21, also known as T-bet, is a transcription factor that plays a role in the regulation of interferon-gamma (IFN-γ) and differentiation of naïve CD4^+^ T cells into Th1 cells. TBX21 can influence Th1/Th2 immune balance by regulating IFN-γ production and Th1 cell differentiation^30,31^. The most pronounced changes in gene expression induced by EEA and PA were identified in type I interferon signaling. In addition, the significant increase in TBX21 gene expression with both EEA and PA treatments was a key finding of these analyses (Fig. 6b,c). In conclusion, our results demonstrated that EEA and PA exhibit promising anti-inflammatory and anti-itch properties in both HMC-1 and HaCaT cell models. This suggests potential applications in the treatment of conditions associated with skin inflammation and itching-regulated respectively.

**Figure 7:**
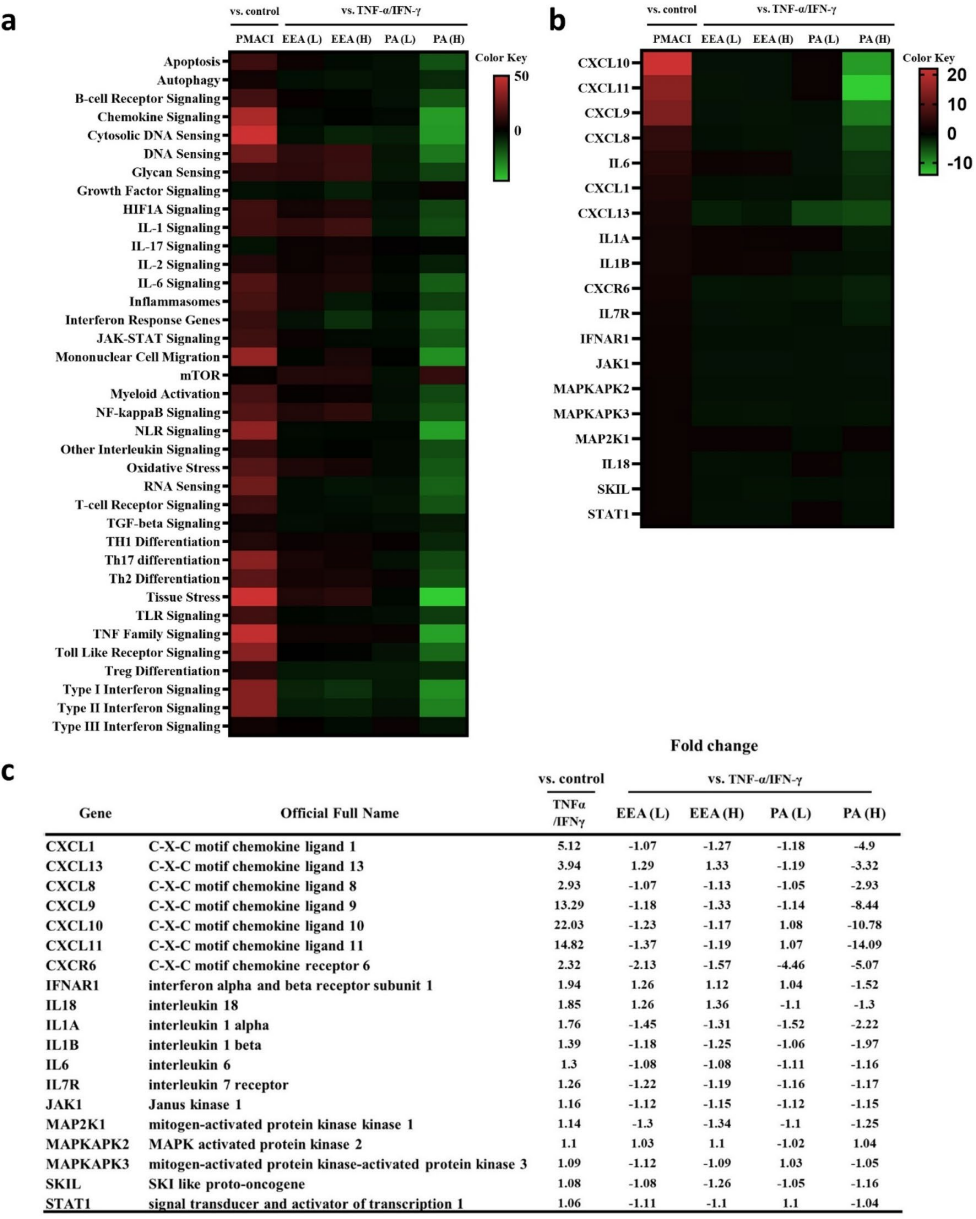
Pathway scoring and gene-set analyses of HaCaT cells. (**a**) 19 genes identically expression in each sample. (**b**) Important pathways between the EEA and PA compared with TNF-α/IFN-γ groups. TNF-α/IFN-γ group vs baseline control group. (**c**) Organized into gene expression table.

The CXCL family, IL family, and signaling molecules are all intricately linked to HaCaT cells and skin inflammation and play pivotal roles in the regulation of immune and inflammatory responses in the skin. The CXCL family, including CXCL1, CXCL8, CXCL9, CXCL10, CXCL11, and CXCL13, plays a crucial role in inflammation and various diseases, including skin-related conditions^32–34^. The results of HaCaT cell experiments revealed significant variations in the expression of signaling-related genes between the TNF-α/IFN-γ-induced positive control and normal control. Notably, the gene expression of cytokines and chemokines (CXCLs, ILs), along with signaling molecules such as JAK1 and MAP2K1, which are implicated in skin inflammation induced by TNF-α/IFN-γ, was substantially diminished following treatment with high concentrations of PA(H) (Fig. 7a–c).

In conclusion, our results demonstrated that EEA exhibits promising anti-inflammatory and anti-itch properties in both HMC-1 and HaCaT cells. This suggests potential applications in the treatment of conditions associated with skin inflammation and itching.

## Discussion

In this study, we investigated the therapeutic potential of *Erigeron annuus* (EA) and its primary active constituent, pyromeconic acid (PA), in managing skin inflammation and itching associated with conditions such as psoriasis, eczema, and atopic dermatitis. Previous studies have mainly focused on the use of topical corticosteroids and antihistamines for treatment, although they are effective to a certain extent, are limited in their efficacy against non-histaminergic itch, and have a risk of side effects such as skin atrophy and hyperpigmentation with prolonged use. We aimed to investigate the antihistaminic, anti-inflammatory, and antioxidant properties of *E*. *annuus* water extract (EEA) and PA. Using both human mast cells (HMC-1) and keratinocytes (HaCaT), our results provide evidence of the efficacy of EEA and PA in alleviating oxidative stress and inflammatory responses, offering a promising plant-based alternative for managing skin health and inflammatory conditions.

In our study, we explored the antioxidant properties of the extract of *Erigeron annuus* without the roots, a deviation from previous research which often used specific plant parts or ethanol extracts. Prior studies have highlighted the presence of phenolic constituents like apigenin, quercetin-3-O-glucoside, and caffeic acid in EA, known for their antioxidant effects^35^. Additionally, phenolic acids in EA have been associated with anti-obesity benefits^14,18,36^. We observed high antioxidant activity in EA, consistent with previous studies. Notably, we used three different extraction methods and found that cold water extraction at 30 °C was most effective for obtaining a high concentration of pyromeconic acid (PA), the primary indicator we analyzed. Our results demonstrated that both *Erigeron annuus* extract (EEA) and pyromeconic acid (PA) significantly mitigated H2O2-induced oxidative stress in skin cells. This was evidenced by improved cell viability, reduced ROS production, and the restoration of key antioxidant enzymes like SOD and CAT. These findings underscore the potent antioxidant capabilities of EEA and PA, offering promising insights into their potential for protecting skin cells from oxidative damage. This study not only reinforces the known antioxidant properties of EA but also expands our understanding of its efficacy, particularly when using whole plant extracts and optimized extraction methods.

In this study, we used HMC-1 and HaCaT cells, which are widely recognized for their importance in studies of cytokines associated with skin inflammation and itching^37,38^. These cell lines are essential for studying factors that induce inflammatory responses, such as histamine secretion from mast cells, a major contributor to inflammation and itching in atopic dermatitis (AD)^39^. The expression of cytokines and chemokines in these cells is closely linked to the inflammatory responses seen in AD. Previous studies have shown that *Sargassum horneri* (Turner) C. Agardh (SHE) shows promising results in reducing inflammation and itching in AD^40^. In addition, studies using HMC-1, HaCaT, and EOL-1 cells have suggested that histamine released from mast cells can exacerbate itch and inflammatory responses^11^. Our study contributes to this knowledge by demonstrating that *Erigeron annuus* extract (EEA) and pyromeconic acid (PA) have significant antihistaminic and anti-inflammatory effects in HMC-1 and HaCaT cells. Specifically, we observed marked inhibition of PMACI-induced cytokine and histamine production. Furthermore, TNF-α/IFN-γ induced pro-inflammatory cytokines (IL-1β and IL-6) and chemokines (TARC and MDC) were effectively reduced by EEA and PA in HaCaT cell. Our findings on the anti-inflammatory and antioxidant effects of EEA and PA are consistent with the documented benefits of *Artemisia herba*-*alba* extracts in modulating glutathione metabolism, resulting in reduced ROS production and enhanced anti-inflammatory effects^41^. These findings underscore the potent anti-inflammatory capabilities of EEA and PA and highlight their potential to alleviate skin inflammation at the molecular level.

Cytokines significantly influence skin barrier regulation^42^, impacting key components like ceramides and barrier proteins such as filaggrin and loricrin^29^. A study demonstrated that oat extract at 0.06% positively affected skin barrier genes (AQP3, FLG, CASP14) and interleukins in HaCaT cells, while also aiding in skin barrier dysfunction recovery^29^. Our study, however, 0.2% EEA and 4.7 µg/mL PA no significant CASP14 expression. This suggests the need for optimizing EEA and PA concentrations and exposure times for desired skin barrier effects. Our findings highlight the complexity of skin barrier modulation and the potential specificity of active ingredients in skincare formulations.

In our skin inflammation study, we observed a significant decrease in the levels of CXCL10, CXCL11, and CXCL9, which are part of the CXC cytokine family and play an important role in immune cell activation and inflammation. This reduction suggests a potential attenuation of the inflammatory response as these chemokines are typically involved in the activation of immune cells in various skin conditions. The reduction in these specific chemokines is consistent with a potential reduction in the severity or extent of inflammation, particularly in conditions driven by autoimmunity, wounds or infectious diseases^33,34,43^.

The transcription factor T-bet, encoded by the TBX21 gene, is known to play a crucial role in the regulation of IFN-γ, a pleiotropic cytokine with antiviral, antitumor, and immunomodulatory functions. In an inflammatory environment, IFN-γ triggers the activation of the immune response and stimulates the elimination of pathogens, while also preventing over-activation of the immune system and tissue damage^44^ TBX21 has also been found to be involved in immunological processes, and increased expression of TBX21 has been associated with better prognosis in skin cutaneous melanoma, they can influence the Th1/Th2 immune balance^31,45^. In our study, we observed a significant upregulation of TBX21, a transcription factor known to promote IFN-γ production, in HMC-1 cells treated with EA water extract (EEA) and PA. Interestingly, despite the increase in TBX21, IFN-induced proteins were simultaneously decreased. This unexpected finding suggests a potential anti-inflammatory effect of EEA and PA, as IFN-γ is typically associated with pro-inflammatory responses and a complex regulatory mechanism is at play, possibly involving other cytokines or signaling pathways. This could be beneficial for skin barrier function as excessive IFN-γ activity is associated with skin barrier dysfunction in conditions such as atopic dermatitis. Our results suggest that EEA and PA can modulate the immune response in a way that reduces inflammation without triggering the harmful effects of excessive IFN-γ. Further research is needed to elucidate the exact pathways involved and to confirm these findings in vivo.

## Materials and methods

### Preparation of *Erigeron annuus* extracts

*Erigeron annuus* (EA) was sourced from Cheongju, Chungcheongbuk-do, Korea. The aboveground parts of the plant, encompassing flowers, leaves, stems, and branches, excluding the roots, were acquired in dried form from Herb Village Co., Ltd., a supplier specializing in raw materials for cosmetics. The identification of the plant material was conducted by Goya Choi, Principal Researcher, Doctor of Korean Medicine/Ph.D. (Herbal Medicine Resources Research Center, Korea Institute of Oriental Medicine, Naju-si, Republic of Korea) and genetic identification was provided (supplementary material, S2). The corresponding voucher specimen (HAM-K043-001) was stored at the Hamsoapharm R&D center. Extraction was performed using purified distilled water at a ratio of 40:1 (water to dried plant material by weight). The mixture was stirred continuously at 30 °C or 90 °C for 8 h. After extraction, the solution was filtered through a 1-micron filter to eliminate particulate matter. After filtration, the extract was concentrated under reduced pressure until a solid content of 10% was obtained (10 Brix). All experiments and procedures comply with the IUCN Policy Statement on Research Involving Species at Risk of Extinction and the Convention on the Trade in Endangered Species of Wild Fauna and Flora.

### Identification and quantification of pyromeconic acid by HPLC

The quantification of pyromeconic acid (PA) in *Erigeron annuus extracts* (cold-, hot water, and ethanol) were conducted using an Agilent 1260 Infinity High-Performance Liquid Chromatography (HPLC) system (Agilent Technologies, Palo Alto, CA, USA), comprising a binary pump, online degasser, autosampler, and Diode Array Detector (DAD). Chromatographic separation was achieved with an Eclipse Plus C18 column (4.6 × 250 mm I.D., 5 µm, Agilent, USA). EEA (5 g of EEA was collected and ultrasonically extracted with 20 mL of 50% methanol for 20 min, followed by 30 mL of 50% methanol, filtered using a 0.45 µm membrane filter, and used as the test solution. The mobile phase consisted of 0.1% H_3_PO_4_ in H_2_O (A) and acetonitrile (B), with a detection wavelength of 270 nm and a flow rate of 0.8 mL/min. The analysis was performed at a controlled temperature of 25 °C using a gradient program that maintained 90% mobile phase A for the first 10 min. For each run, 10 µL of both the standard and test solutions was injected into the HPLC system.

### Measurement of DPPH and ABTS radical-scavenging activity

To assess the antioxidant capacity of the EEA extract, a DPPH (2,2-diphenyl-1-picrylhydrazyl) radical scavenging assay was conducted with slight modifications to the method described in a previous study^46^. Briefly, a DPPH solution was prepared by dissolving 0.1 mM DPPH in methanol. The 0.05 mL of EEA extract (0.05 mL (1:1, v/v)) was mixed with 0.1 mM DPPH solution. The mixture was incubated for 30 min at room temperature in the dark. The absorbance was measured at 517 nm using a spectrophotometer. The radical scavenging activity of the EEA extract was calculated, expressed as a percentage, and compared with that of the control methanol and standard ascorbic acid.

The ABTS assay was performed to measure the antioxidant activity as follows: First, 7 mM ABTS solution (Sigma-Aldrich, A1888) was prepared in distilled water and stored at 4 °C. Next, a 245 mM APS solution (Sigma-Aldrich, A3678-25G) was prepared in ultrapure water. To generate ABTS radicals, APS was added to the ABTS solution to a final concentration of 2.45 mM. The mixture was incubated overnight at room temperature in the dark for 16 h. The concentration of ABTS radical solution was measured at a wavelength of 734 nm. A solution of 10 mM Trolox (Sigma-Aldrich, 238813) in methanol was prepared. Next, 10 μL of sample or standard (Trolox) was mixed with 190 μL of ultrapure water in a well of a microplate. Blanks used methanol for standards and distilled water for samples. The mixtures in the wells were vortexed, and incubated in the dark for 5 min, and the absorbance was measured at 734 nm.

### Intracellular ROS measurement

The concentration of intracellular ROS was measured by means of a carboxy-H2DCFDA probe. HaCaT cells (2 × 10^4^ cells/cm^2^) were seeded in 6-well cell culture plates for 24 h, pretreated with EEA (0.1% or 0.2%) and PA (respective concentrations, 4.7 and 9.4 µg/mL) for 6 h and stimulated with 250 µM H_2_O_2_ for 12 h. The cells were further incubated with 10 µM carboxy-H_2_DCF-DA for 20 min. After incubation, the cells were washed with 1× PBS, and images were captured using an Olympus IX53 microscope (Olympus).

### Measurement of lipid peroxidation and antioxidant enzymes

To investigate the effects of EEA on oxidative stress responses, HaCaT cells were pre-treated with varying concentrations of EEA for 6 h, and then, hydrogen peroxide (H_2_O_2_) for 12 h to induce oxidative stress. Cells were then harvested for further analysis. Total malondialdehyde (MDA), superoxide dismutase (SOD), and Catalase Activity (CAT) assay were performed according to manufactural’s of DogenBio (Seoul, Korea).

### Cell culture conditions

Human epidermal keratinocytes (HaCaT) cells were cultured in Dulbecco's modified Eagle's medium (DMEM, LM001-05; Welgene) supplemented with 10% fetal bovine serum (FBS, Gibco, Carlsbad, CA, USA), 1% penicillin and streptomycin (P/S, GIBCO) at 37 °C in a humidified chamber with 5% CO_2_. Human mast cell (HMC-1) cells were cultured under the same conditions, using Iscove's modified Dulbecco's medium (IMDM, LM004-01; Welgene).

### Cell viability assay

HaCaT and HMC-1 cell viability was measured using the EZ-Cytox reagent (EZ-1000, Dogenbio). The HaCaT cells (1 × 10^4^ cells/cm^2^) and HMC-1 (1 × 10^5^ cells/mL) were seeded in a 96-well plate for 24 h. For the HMC-1 cells, due to the characteristics of the suspension cells, the treatment of the samples was started immediately 30 min after seeding. The cells were then pretreated with different concentrations (0.05–1%, concentrations of extracts show diluted content based on 10 brix of EEA) of EEA for 24 and 48 h. After incubation, 0.01 mL EZ-Cytox reagent was added to each well and incubated for 2 h. The absorbance in each well was then measured at 450 nm using a microplate reader.

### Establishment of cell models for inflammation, and skin barrier damage

To induce an inflammatory response in the cells, briefly, HMC-1 cells were treated with a mixture of 50 nM PMA and 1 µM A23187 (PMACI) and induced with 24 h PMACI after 1 h of EEA and PA pretreatment; for histamine, we measured the inhibition of histamine secretion after 1, 6, and 12 h of EEA and PA pretreatment respectively. HaCaT cells were induced with a mixture of 10 ng/mL TNF-α and 10 ng/mL IFN-γ to induce an inflammatory response, which was induced by 24 h of treatment after 6 h of sample pretreatment. To establish a cell model of skin barrier dysfunction, we referred to previous study^29^. Various concentrations of sodium dodecyl sulfate (SDS) were treated for 24 h and the concentration that 60% of the cell viability was selected and subsequently treated for 24 h after 6 h pre-treatment of the EEA and PA to construct a skin barrier dysfunction model.

### Real-time quantitative PCR

Total RNA from cells was isolated using Quiazol and AccuPrep^®^ Universal RNA Extraction Kit (K-3140, Bioneer) according to the manufacturer’s guidelines. cDNAs were generated from 1 µg of total RNA using the iScript cDNA Synthesis Kit (#1708890, Bio-Rad). Gene expression levels were evaluated by Real-time qPCR using a StepOnePlus (Applied Biosystems) and normalized to GAPDH (Housekeeping gene) gene expression. The primer sequences are collected in Supplementary Table 1.

### ELISA

Cell culture supernatant was centrifuged at 10,000×*g* at 4 °C for 20 min and collected the following experiments. The concentration of histamine (ab213975, Abcam), IL-6, IL-1β, IFN-γ, TARC, and MDC in cell culture supernatant was determined using ELISA kits (Proteintech) according to the manufacturer's recommendations.

### NanoString analysis

The gene expression analysis was performed at PhileKorea Technology (Daejeon, Korea) on a NanoString nCounter gene expression platform by Auto Immune profiling panel and Host Response advanced panel (NanoString Technologies, WA, USA). A custom code set consisting of a 773 gene panel related to infectious diseases, and 56 pathway in Host response panel, and a 750 gene panel related to autoimmune and autoinflammatory diseases in Auto Immune profiling panel was used in this study. nSolver (version 4.0, NanoString Technologies) was used for differential gene expression analysis of nCounter analysis data. All reporter code count files passed the default QC settings, and by default, all raw gene expression data was normalized in the Positive Control and Housekeeping normalization steps. Fold changes were calculated by averaging normalized lanes from controls.

### Statistical analysis

We statistically analyzed all data using GraphPad Prism (version 10.1.0). The real-time qPCR and ELISA results of the differences between groups were determined using one-way analysis of variance and Dunnett’s multiple comparisons test, and all data were independently replicated in triplicate. *P ≤ 0.05 was the statistically significant difference between the group of normal control and positive control (stimuli control). ^#^P ≤ 0.05 was the statistically significant difference between the group of positive control (stimuli control) and EEA or PA. Cell viability assay was performed using the Unpaired t-test. All of the data from the Nanostring nCounter platform was analyzed and compared using the nSolver analysis software, such as QC, normalization, gene expression, and Heatmap.

### Supplementary Information

Supplementary Information.

## Data Availability

The corresponding authors can grant datasets formed and sifted in this study upon sanity demand.

## References

[1] Kaaz K, Szepietowski JC, Matusiak Ł (2019). Influence of itch and pain on sleep quality in atopic dermatitis and psoriasis. Acta Derm. Venereol..

[2] Ikeda Y, Murakami A, Ohigashi H (2008). Ursolic acid: An anti-and pro-inflammatory triterpenoid. Mol. Nutr. Food Res..

[3] Yosipovitch G (2019). Skin barrier damage and itch: Review of mechanisms, topical management and future directions. Acta Derm. Venereol..

[4] Langan SM, Abuabara K, Henrickson SE, Hoffstad O, Margolis DJ (2017). Increased risk of cutaneous and systemic infections in atopic dermatitis—A cohort study. J. Investig. Dermatol..

[5] Harrison IP, Spada FJM (2019). Breaking the itch–scratch cycle: Topical options for the management of chronic cutaneous itch in atopic dermatitis. Medicines (Basel).

[6] Aliotta, G. E. Characterization and modulation of histaminergic and non-histaminergic itch (2022).

[7] Sutaria N (2022). Itch: Pathogenesis and treatment. J. Am. Acad. Dermatol..

[8] Gilchrist M, Befus AJI (2008). Interferon-γ regulates chemokine expression and release in the human mast cell line HMC1: Role of nitric oxide. Immunology.

[9] Thangam EB (2018). The role of histamine and histamine receptors in mast cell-mediated allergy and inflammation: The hunt for new therapeutic targets. Front. Immunol..

[10] Sellge G (2014). Interferon-γ regulates growth and controls Fcγ receptor expression and activation in human intestinal mast cells. BMC Immunol..

[11] Oh JS, Seong GS, Kim YD, Choung SY (2021). Effects of deacetylasperulosidic acid on atopic dermatitis through modulating immune balance and skin barrier function in HaCaT, HMC-1, and EOL-1 cells. Molecules.

[12] Zhang Y (2022). Skin care product rich in antioxidants and anti-inflammatory natural compounds reduces itching and inflammation in the skin of atopic dermatitis patients. Antioxidants.

[13] Sheet, P. F. & Page, G. C. *Erigeron**philadelphicus* L.

[14] Zhang L (2020). Antioxidant and enzyme-inhibitory activity of extracts from *Erigeron*
*annuus* flower. Ind. Crops Prod..

[15] Jo MJ (2013). Roots of *Erigeron*
*annuus* attenuate acute inflammation as mediated with the inhibition of NF-κB-associated nitric oxide and prostaglandin E2 production. Evid. Based Complement. Alternat. Med..

[16] Rana R (2023). Phytochemistry and biological activity of *Erigeron*
*annuus* (L.) Pers. Naunyn Schmiedebergs Arch Pharmacol.

[17] Jeong M, Kwon H, Ju Y, Choi G-E, Hyun K-Y (2022). Protective effect of aqueous extract from *Erigeron*
*annuus* against cell death induced by free radicals. Biomed. Sci. Lett..

[18] Zheng Y, Choi YH, Lee JH, Lee SY, Kang IJ (2021). Anti-obesity effect of *Erigeron*
*annuus* (L.) Pers. extract containing phenolic acids. Foods.

[19] Rana R (2023). Phytochemistry and biological activity of *Erigeron*
*annuus* (L.) Pers. Naunyn Schmiedebergs Arch. Pharmacol..

[20] Joo D-H, Yoo D-H, Lee J-Y (2019). A study on the whitening effect of *Erigeron*
*annuus* (L.) Pers. ethanol extract on melanoma cell (B16F10). Micrbiol. Biotechnol. Lett..

[21] Guarneri F, Bertino L, Pioggia G, Casciaro M, Gangemi SJA (2021). Therapies with antioxidant potential in psoriasis, vitiligo, and lichen planus. Antioxidants.

[22] Soares GB, Mahmoud O, Yosipovitch GJI (2023). Role of antioxidants in itch treatment: Lessons learned from pain management. Itch.

[23] Aliahmat NS (2012). Antioxidant enzyme activity and malondialdehyde levels can be modulated by *Piper*
*betle*, tocotrienol rich fraction and *Chlorella*
*vulgaris* in aging C57BL/6 mice. Clinics.

[24] Zhang J, Wang W, Mao X (2020). Chitopentaose protects HaCaT cells against H2O2-induced oxidative damage through modulating MAPKs and Nrf2/ARE signaling pathways. J. Funct. Foods.

[25] Park H-H (2007). Anti-inflammatory activity of fisetin in human mast cells (HMC-1). Pharmacol. Res..

[26] Fu L (2021). Interleukin-35 inhibited the production of histamine and pro-inflammatory cytokines through suppression MAPKs pathway in HMC-1 cells. Allergy Asthma Clin. Immunol..

[27] Fujii-Maeda S (2004). Reciprocal regulation of thymus and activation-regulated chemokine/macrophage-derived chemokine production by interleukin (IL)-4/IL-13 and interferon-γ in HaCaT keratinocytes is mediated by alternations in E-cadherin distribution. J. Investig. Dermatol..

[28] Park J-W (2021). 3, 4, 5-Trihydroxycinnamic acid exerts anti-inflammatory effects on TNF-α/IFN-γ-stimulated HaCaT cells. Mol. Med. Rep..

[29] Ding W, Fan L, Tian Y, He C (2022). Study of the protective effects of cosmetic ingredients on the skin barrier, based on the expression of barrier-related genes and cytokines. Mol. Biol. Rep..

[30] Leng R-X (2016). Evidence for genetic association of TBX21 and IFNG with systemic lupus erythematosus in a Chinese Han population. Sci. Rep..

[31] Iwata S (2017). The transcription factor T-bet limits amplification of type I IFN transcriptome and circuitry in T helper 1 cells. Immunity.

[32] Lu J, Chatterjee M, Schmid H, Beck S, Gawaz M (2016). CXCL14 as an emerging immune and inflammatory modulator. J. Inflamm..

[33] Chen X, Chen R, Jin R, Huang Z (2020). The role of CXCL chemokine family in the development and progression of gastric cancer. Int. J. Clin. Exp. Pathol..

[34] Zhou C, Gao Y, Ding P, Wu T, Ji G (2023). The role of CXCL family members in different diseases. Cell Death Discov..

[35] Lee HJ, Seo Y (2006). Antioxidant properties of *Erigeron*
*annuus* extract and its three phenolic constituents. Biotechnol. Bioprocess Eng..

[36] Jeong C-H (2011). Neuroprotective and anti-oxidant effects of caffeic acid isolated from *Erigeron*
*annuus* leaf. Chin. Med..

[37] Theoharides TC, Kalogeromitros D (2006). The critical role of mast cells in allergy and inflammation. Ann. N. Y. Acad. Sci..

[38] Benezeder T (2020). Dithranol targets keratinocytes, their crosstalk with neutrophils and inhibits the IL-36 inflammatory loop in psoriasis. Elife.

[39] Lee K-S (2018). The prevention of TNF-α/IFN-γ mixture-induced inflammation in human keratinocyte and atopic dermatitis-like skin lesions in Nc/Nga mice by mineral-balanced deep sea water. Biomed. Pharmacother..

[40] Dias MKHM (2021). *Sargassum*
*horneri* (Turner) C. Agardh ethanol extract attenuates fine dust-induced inflammatory responses and impaired skin barrier functions in HaCaT keratinocytes. J. Ethnopharmacol..

[41] Bouchara N (2021). Anti-inflammatory and prolonged protective effects of *Artemisia*
*herba*-*alba* extracts via glutathione metabolism reinforcement. S. Afr. J. Bot..

[42] Hänel KH, Cornelissen C, Lüscher B, Baron JM (2013). Cytokines and the skin barrier. Int. J. Mol. Sci.

[43] Kuo PT (2018). The role of CXCR3 and its chemokine ligands in skin disease and cancer. Front. Med..

[44] Jorgovanovic D, Song M, Wang L, Zhang Y (2020). Roles of IFN-γ in tumor progression and regression: A review. Biomark. Res..

[45] Zhang X (2020). Increased expression of T-box transcription factor protein 21 (TBX21) in skin cutaneous melanoma predicts better prognosis: A study based on the cancer genome atlas (TCGA) and genotype-tissue expression (GTEx) databases. Med. Sci. Monit..

[46] Ogliani E, Skov AL, Brook MAJAMT (2019). Purple to yellow silicone elastomers: Design of a versatile sensor for screening antioxidant activity. Adv. Mater. Technol..

