# Supplementary material for: Pyromeconic acid-enriched Erigeron annuus water extract as a cosmetic ingredient for itch relief and anti-inflammatory activity
Source: Sci Rep. 2024 Feb 26;14:4698. doi: 10.1038/s41598-024-55365-2 (PMC10897215; doi:10.1038/s41598-024-55365-2)
Supplement: Supplementary file 1 — Supplementary Information. [file 41598_2024_55365_MOESM1_ESM.docx]

**<SUPPORTING INFORMATION>**

**Pyromeconic Acid-Enriched *Erigeron annuus* Water Extract as a Cosmetic Ingredient for Itch Relief and Anti-Inflammatory Activity**

Minkyoung Kang^1^, Minji Kang^1^, Tae Hee Kim^2^, Seong Un Jeong^2^, and Sangnam Oh^1*^

*^1^Department of Functional Food and Biotechnology, Jeonju University, Jeonju 55069, Korea*

***^2^Hamsoapharm R&D Center, Korea***

*To whom correspondence should be addressed: osangnam@jj.ac.kr

**S1.** List of primers used in this study for Real-Time-quantitative PCR

| Primer | Sequence (5’-3’) | Annealing temperature (℃) |
| --- | --- | --- |
| IL-1β | Forward: ACGCTCCGGGACTCACAGCA | 60 |
|  | Reverse: TGAGGCCCAAGGCCACAGGT |  |
| IL-6 | Forward: CCTTCGGTCCAGTTGCCTTCT | 60 |
|  | Reverse: CAG TGC CTC TTT GCT GCT TTC |  |
| IFN-γ | Forward: TGACCAGAGCATCCAAAAGA | 55 |
|  | Reverse: CTCTTCGACCTCGAAACAGC |  |
| TARC | Forward: CGGACCCCAACAACAAGAGA | 60 |
|  | Reverse: AGTCAGGAGTCTGGGGTGAG |  |
| MDC | Forward: CTACTCTGATGACCGTGGCC  Reverse: AGGGAATGCAGAGAGTTGGC | 60 |
| FLG | Forward: TGGCAGCTATGGTAGTGCAG  Reverse: TGGCCACATAAACCTGGGTC | 60 |
| CASP14 | Forward: GAAATCCAAAGCACCCTCCG  Reverse: GACCTGCATGAAGGGTGTGA | 60 |
| GAPDH | Forward: CAACGACCACTTTGTCAAGC  Reverse: TTCCTCTTGTGCTCTTGCTG | 60 |

* FLG; Filaggrin, CASP14; Caspase 14

S2. Analysis of Clustal Multiple Alignment in Internal Transcribed Spacer(ITS)


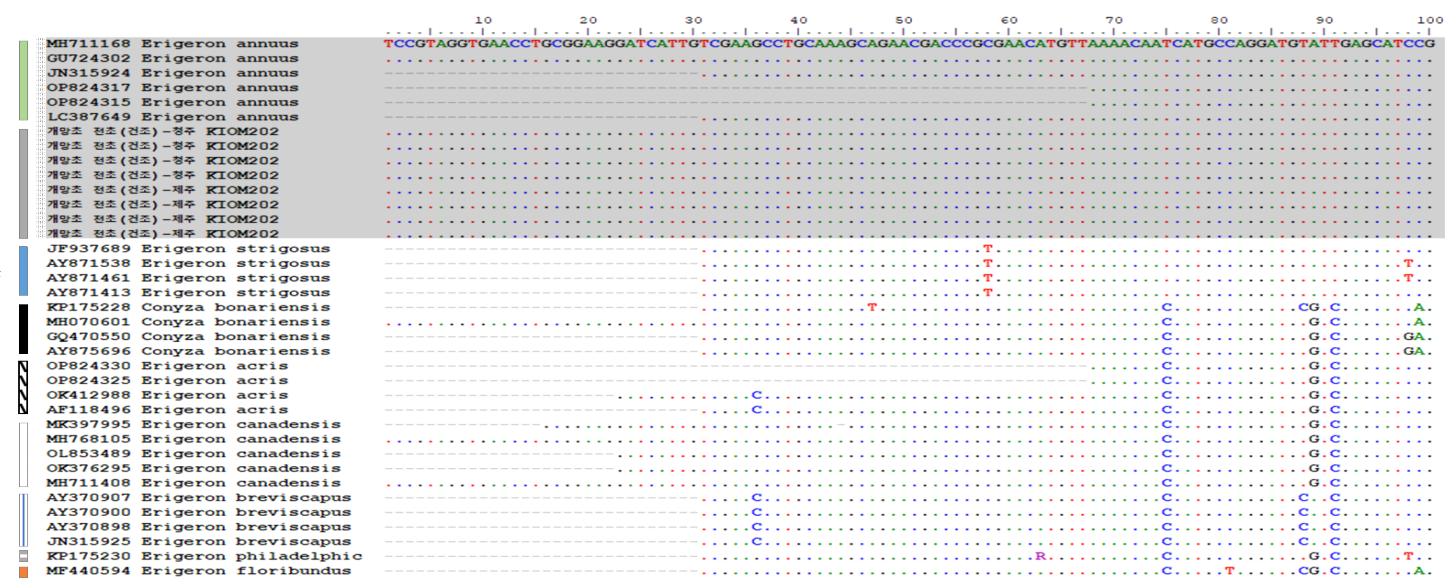


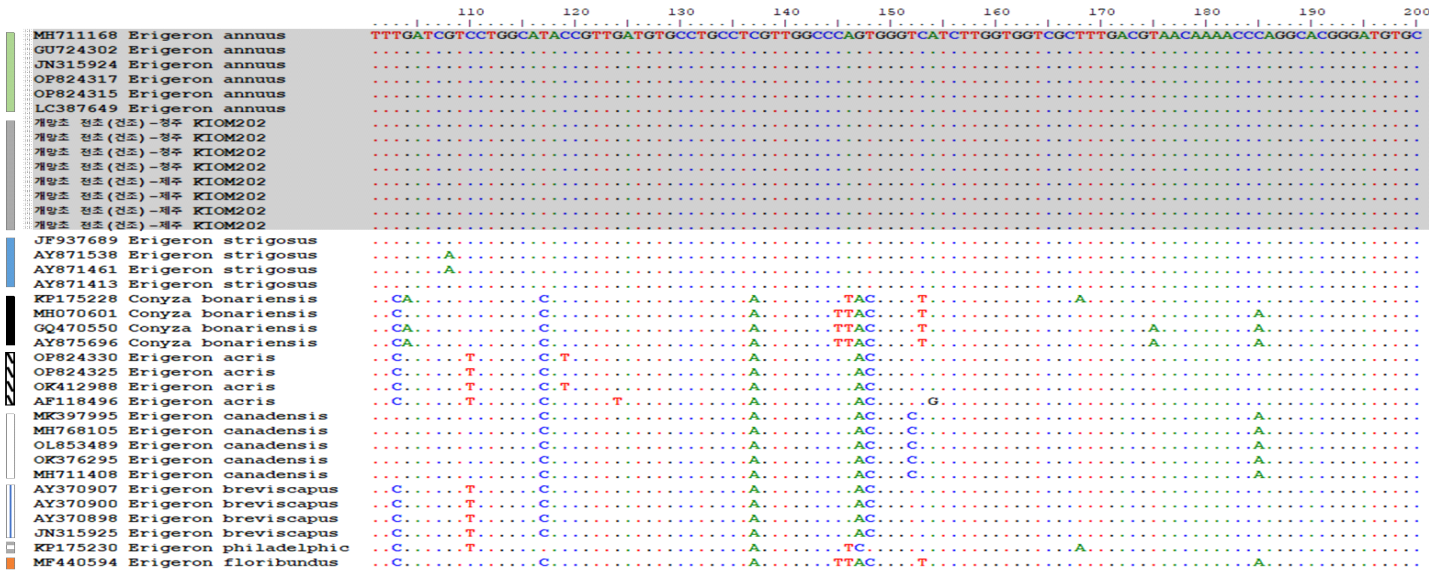


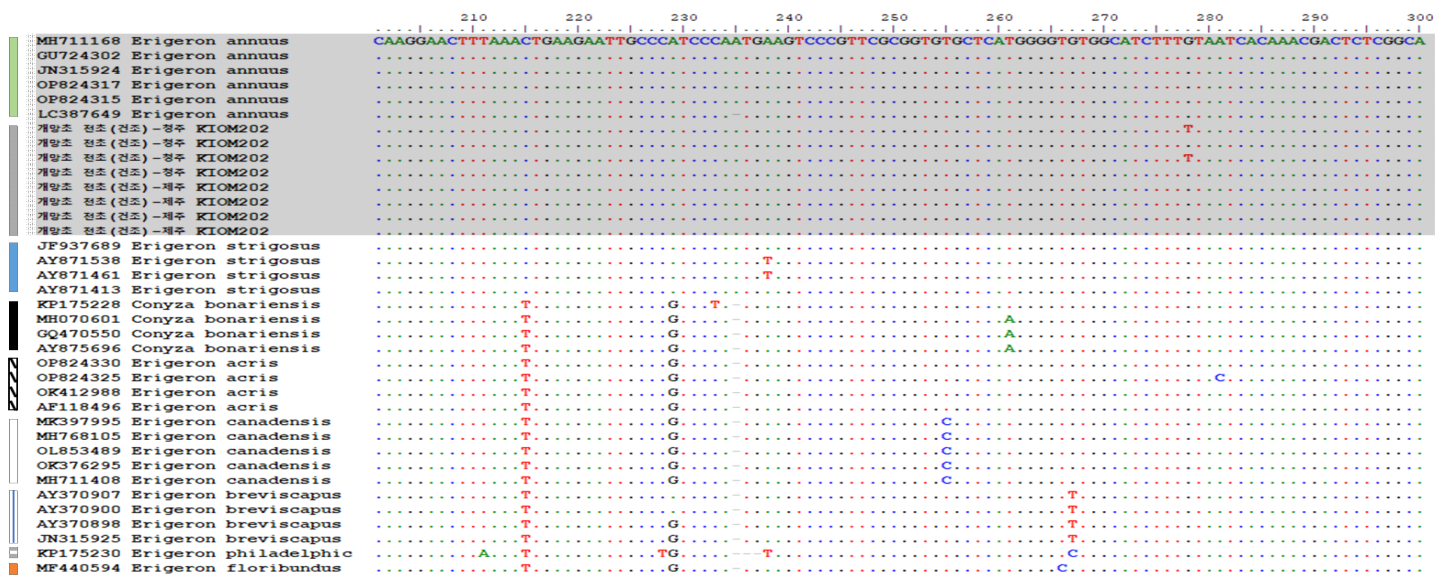


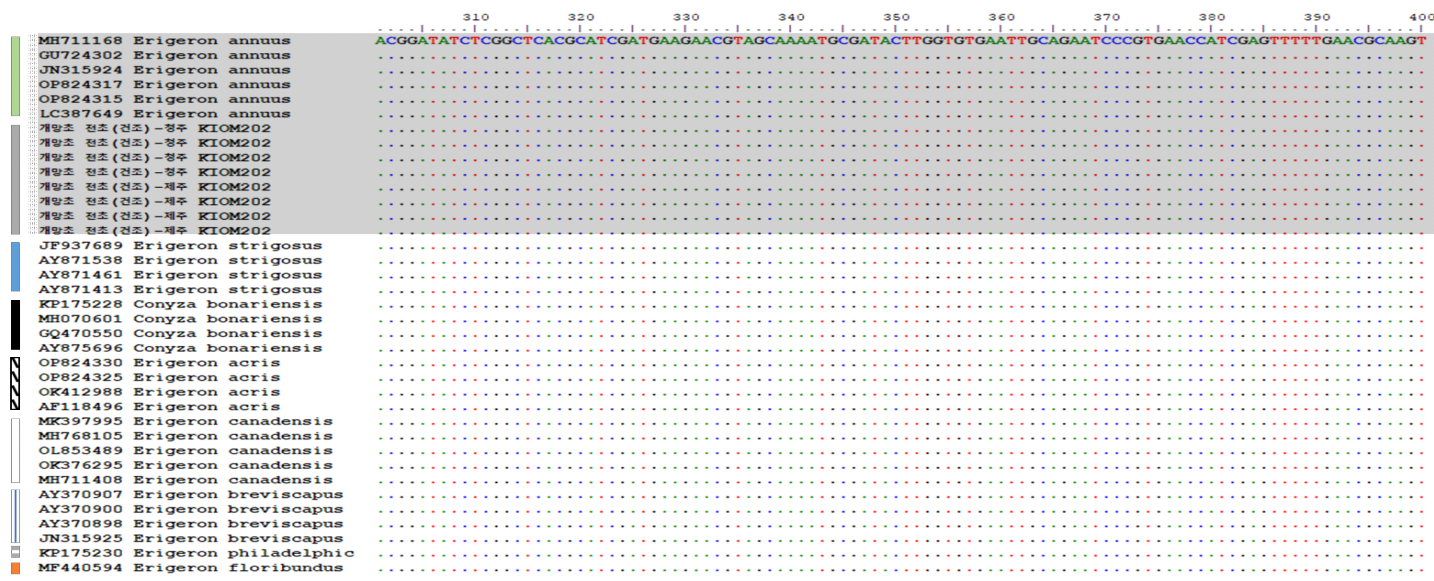


* Requested sample name: KIOM202

* Gray colored box shows KIOM202 samples are identified as *Erigeron annus*
